# Case of Dislocation of Femur, Reduced by Dr. Reid’s Method

**Published:** 1852-07

**Authors:** J. H. Beech

**Affiliations:** Cold Water, Michigan


ART. III.—*Case of Dislocation of the Femur, reduced by Dr. Reid's method.* By J. H. BEECH, M. D., Cold Water, Michigan.

I am induced to send you the following report of a case of dislocation of the head of the femur downward and backward: first, because of the rarity of this accident; and secondly, on account of the perfect adaptation of Dr. Reid's femoral lever to the reduction of this luxation:

March 28th, 1852. Was called into the house of Mr. Samuel Otis, about noon, to see his son, aged two years and one month. Learned that, the day before, he was on his hands and knees, when his brother, two and a quarter years older, jumped suddenly upon his back, by which he was instantly brought to the floor upon his face and left shoulder, with the left leg under him. His screams were violent, and continued to be so whenever the limb was moved upon the body. The mother was obliged to keep him in her arms in a partially flexed position most of the time, and even then he seemed to suffer considerably.

I found the thigh slightly swollen; the toes inverted; and the limb half an inch longer than the sound one, when both were made straight, which gave great pain.

When laid upon the back with the thighs at right angles with the body, the knee was more than half an inch lower than the sound one. Laying upon the face, the body upon a pillow, the trochanter major was found farther back than the tuber ischii, and farther from the crest of the ilium, and also, the flatness between the crest and the trochanter, contrasted strongly with the roundness of the other side.

There was no crepitation, nor any difference in the length of the femurs that I could discern. My diagnosis was, of course, dislocation of the head of femur upon the spine of the ischium, or into the lesser ischiatic notch. From the violence of his cries during the examination, I thought an anæsthetic advisable, but as he resisted the inhalation with great energy, it was not persevered in, thinking better to try, first, "Dr. Reid's method."

Accordingly, tke little patient was laid on his back upon a hard bed, with the shoulders confined by the mother, and the pelvis and right leg held firm by the father, while I proceeded to flex the thigh upon the body, and the leg upon the thigh, (allowing the toes to take their own direction, that of inversion,) carrying the knee over the right thigh in its passage upward. When a little higher than at right angle, I was confident that I felt the head of the femur come in contact with the edge of the acetabulum, upon which,

I increased the adduction, and continued the flexion, bringing the knee pretty firmly upon the body, and then allowed it to move outward, upon which a sensation of gentle crushing, or sliding, was felt, and the patient altered the tone of his cries very perceptibly. The limb was now brought flat upon the bed, and all restraint removed. The position of the toes, and length of the limb, were found to correspond precisely with the sound one, and after remaining quiet a moment he asked to be taken up, but made no complaint as before, on being moved. He now sat erect on the lap with both hip and knee joints at right angles, and said cheerfully, "I aint sick now."

The reduction did not take half as long, and seemed no more painful than my examination had been. My own feelings were so much like "extacy" in the conclusion, that I was "very much obliged" *to Dr. Reid*, notwithstanding all his competitors for the honor of priority in the use of the shaft of the femur as a lever to reduce luxations of the head upward, &c.

If my letter has not become too lengthy, it may possess enough practical interest to be laid before the professional readers. It is most respectfully submitted to your judgment, and I shall not object to seeing it condensed or clipped with severity provided verity prevails. Perhaps I should add that,

March 30. Patient was comfortable; leg some swollen; has tried to step on it but cried from pain.

May 17. The father says the boy has been as well as ever for some time.

